# Epidemiological characteristics and societal burden of varicella zoster virus in the Netherlands

**DOI:** 10.1186/1471-2334-12-110

**Published:** 2012-05-10

**Authors:** Jorien GJ Pierik, Pearl D Gumbs, Sander AC Fortanier, Pauline CE Van Steenwijk, Maarten J Postma

**Affiliations:** 1 Department of Pharmacy, Unit of PharmacoEpidemiology & PharmacoEconomics, University of Groningen, Antonius Deusinglaan 1, Groningen 9713 AV, The Netherlands; 2 Health Technology & Services Research, MIRA institute for Biomedical Technology and Technical Medicine, University Twente, Drienerlolaan 5, Enschede, 7522 NB, The Netherlands; 3 Corporate Affairs Department, GlaxoSmithKline BV, Huis ter heideweg 62, Zeist, 3705 LZ, The Netherlands; 4 Medical Affairs Department, GlaxoSmithKline BV, Huis ter heideweg 62, Zeist, 3705 LZ, The Netherlands; 5 Department of Medical Informatics, Zorggroep Almere, Randstad 22-01, Almere, 1316 BN , The Netherlands

**Keywords:** Varicella, Herpes zoster, Epidemiology

## Abstract

**Background:**

Varicella and herpes zoster are both caused by varicella zoster virus (VZV) infection or reactivation and may lead to complications associated with a (severe) societal burden. Because the epidemiology of VZV-related diseases in the Netherlands remains largely unknown or incomplete, the main objective of this study was to study the primary care incidence, associated complications and health care resource use.

**Methods:**

We investigated the incidence of VZV complications in the Dutch general practitioner (GP) practices and pharmacies in a retrospective population-based cohort study (2004–2008) based on longitudinal GP data including free text fields, hospital referral and discharge letters from approximately 165,000 patients.

**Results:**

The average annual incidence of varicella GP-consultations was 51.5 per 10,000 (95% CI 44.4-58.7) overall; 465.5 per 10,000 for 0–1 year-olds; 610.8 per 10,000 for 1–4 year-olds; 153.5 per 10,000 for 5–9 year-olds; 8,3 per 10,000 for >10 year olds. When only ICPC coded diagnoses were analyzed the incidence was 27% lower. The proportion of complications among varicella patients was 34.9%. Most frequently complications were upper respiratory tract infections. Almost half of the varicella patients received medication. The referral rate based on GP consultations was 1.7%. The average annual incidence of herpes zoster GP-consultations was 47.5 per 10,000 (95% CI 40.6-54.4). The incidence increased with age; 32.8 per 10,000 for <60 year-olds; 93.1 per 10,000 for 60–64 year-olds and 113.2 per 10,000 for >65 year olds. When estimating herpes zoster incidence only on ICPC coded information, the incidence was 28% lower. The complication rate of herpes zoster was 32.9%. Post herpetic neuralgia was seen most often. Of patients diagnosed with herpes zoster 67.8% received medication. The referral rate based on GP consultations was 3.5%.

**Conclusions:**

For varicella the highest incidence of GP-consultations was found in 1–4 year-olds, for herpes zoster in the >65 years olds. The occurrence of complications was not age-dependent but varies per complication. When estimating incidence of VZV-related diseases in primary care, based on diagnostic codes only, one should be aware of a gross underestimation of the incidence. Our analysis may have important implications for the outcomes of upcoming cost-effectiveness analyses on VZV vaccination.

## Background

Varicella zoster virus (VZV) infection causes two diseases; varicella and herpes zoster. The epidemiology and burden of both VZV-related diseases and associated complications in the Netherlands is largely unknown or incomplete.

The primary disease burden of VZV infection is varicella. Varicella is a common viral childhood disease affecting nearly the entire birth cohort. In a non-vaccinated population such as the Netherlands the risk of acquiring a primary VZV infection is over 97% and most people contract varicella before the age of five [1]. While varicella is usually self-limiting, a case of varicella can potentially develop serious complications and may lead to hospitalization and even death [2-4]. Following primary infection, VZV becomes latent in the dorsal root ganglia and may reactivate later in life resulting in herpes zoster [5]. Herpes zoster is usually a self-limiting vesicular rash, accompanied by it’s most common complication post-herpetic neuralgia (PHN) [6]. But herpes zoster can give rise to other complications, many of which have unusual presentations and serious or even life-threatening sequelae [7,8].

VZV-related incidences and complications have been estimated in different national and international studies [1,2,9-13]. Most of these studies were limited to hospitalization records associated with varicella or herpes zoster as a measure of disease complications. These restrictions do not allow for a full analysis of the complications associated with VZV as most patients are likely to visit only a general practitioner (GP). Therefore, morbidity presented in the general daily practice might be a better indicator of VZV morbidity in the general population [14]. In particular, in the Netherlands GPs are usually the first point of contact with the Dutch healthcare system and basically all non-institutionalized people are registered at a GP.

In the Netherlands, the VZV-incidence presented in the primary care are coded according to the International Classification of Primary Care (ICPC). The Netherlands Institute of Health Services Research (NIVEL) collects weekly these ICPC-coded anonymized patient information on VZV-infection. However, this method comprises a risk of potential underdetection of the disease and related complications because it excludes the analysis of free text fields which allow for a more elaborate analysis of non-coded information.

Prevention by vaccination could be an optimal approach in the management of VZV disease. At this moment decisions about introducing VZV vaccination in routine immunization programme in the Netherlands have not been made, assumed due to the lack on epidemiologic information and perception that varicella in young children may generally not be a serious disease. Given the lack of data, more insights into the epidemiology of VZV infections and into the disease burden, are needed. In particular in the Netherlands, the Dutch Health Council stated that more epidemiological information is required to decide on the introduction of VZV vaccination in the NIP [15]. We studied the primary care incidence of varicella and herpes zoster, their associated complications, health care resource use, in a GP’s research database which also included free text fields.

## Methods

### Setting

Primary health care use was estimated from the Zorggroep Almere (ZGA). Data available through ZGA are based on electronic medical records from 22 general practices, spread throughout Almere, one of the largest Dutch cities (approximately 185,000 inhabitants). ZGA database contains information on approximately 167,000 inhabitants. Data include longitudinal information on patient’s characteristics, such as age, sex, as well as medical information, on consultations, prescriptions, referrals, laboratory values and diagnoses. GPs participating in ZGA are instructed to use ICPC-1 codes for every patient contact. Furthermore, summaries of the hospital discharge letters and information from specialists are entered in a free text format. Information on drug prescription comprises brand name, quantity, strength, indication, prescribed daily dose, dispensing date and the Anatomical Therapeutical Chemical (ATC) classification code.

### Study design

The design of the study was a longitudinal retrospective population-based cohort study. This study was restricted to the analysis of all persons with permanent registration status with one of the GPs who contributed to the database of Zorggroep Almere. In particular, the database comprised 164,631 in 2004 and increased to 166,616 persons in 2008. Based on ICPC-codes A72 and S70, including free text in the notes of the electronic record, probable cases of VZV-infections from January 1st, 2004 to December 31st, 2008 were identified. Note, anonymonized patient data was provided by ZGA, therefore, according to Dutch regulations no ethical approval was required for this study [16].

### Patient identification - case definition

Using a computerized algorithm, we included patients with an ICPC-code for varicella (A72). In addition, to avoid potential underdetection of varicella, we included all patients with suspected varicella, using a sensitive search algorithm to the s section of each electronic medical record. Of all varicella suspected patients with an insect bite or sting (ICPC-code S12) were excluded from our analyses. Herpes zoster patients were included according to the ICPC-code S70 or/and if herpes zoster was suspected. Cases of a previous diagnosed and ongoing treatment for PHN (coded with ICPC-code S70.02) were excluded. Both searches included possible misspellings of varicella and herpes zoster. The computerized medical records of all potential cases were reviewed carefully. We included cases when the records stated an infection with varicella or herpes zoster. For each case, the date of the first VZV-related consultation was taken as index date. For a subgroup of patients the index date was restricted to the month of the GP visit. Considering the feasibility of the analyses we set the date for this group on the first day of the month. Varicella and herpes zoster monthly incidence was extracted from the dataset.

### Definitions of VZV- related complications and symptoms

First, based on a literature search a list of potential medical and lay names for the retrieval of potential cases of VZV-related complications and symptoms from the free text search in the electronic patient records was generated [17-20]. This list was compiled by sending the list of selected complications to a small group of Dutch experts consisting of two GPs and two paediatric infectious disease specialists.

In order to identify VZV-related complications in the GP practice, we performed an extended computerized database search within the free text or on ICPC-code of the medical records using the Dutch version of the words as shown in Additional file 1 (varicella) and Additional file 2 (herpes zoster). Varicella-related complications and symptoms were divided in groups as follow: upper respiratory tract; ENT (ear, nose and throat) and eye complications; lower respiratory tract complications; skin infectious/cutaneous complications; neurologic complications; gastrointestinal tract complications; haematological complications; complications due to (systemic) bacterial infections; and symptoms as a more general category.

Accordingly, reported herpes zoster-related complications and symptoms were categorized into the following ten groups: upper respiratory tract; ENT complications; eye complications; lower respiratory tract complications; skin infectious/cutaneous complications; PHN; neurologic complications other than PHN; gastrointestinal tract complications; haematological complications; complications due to (systemic) bacterial infections; and again symptoms as a more general group. VZV-related complications were defined as those occurring within 30 days before or after consultation for VZV infections, with the exception of post-herpetic neuralgia (PHN), because some cases of PHN may last for several months or years [20]. If the cases visited the GP more than once for the same indication we counted the complication once.

All referrals and discharge letters in the primary care database were reviewed in order to identify VZV-related complications in hospital care among the previously selected VZV-patients between 21 days before and 40 days after the index date. Note, that data was only derived in a primary care setting. We identified all the patients with an ICPC-code for varicella (A72) or herpes zoster (S70) and free text (chickenpox; varicella) or (shingles; herpes zoster) in their referral or discharge letter. To avoid underestimation, all referrals and discharge letters of VZV-cases were reviewed on highly plausible associated complications, for example post-viral cerebellitis. Referral rates were calculated based on GP consultations.

### Treatment of VZV-infections

VZV-related medication use was identified by an extended computerized database search on ATC codes as shown in Additional file 3 . Medication use was selected 21 days before and after the index date and was analyzed on relevance. Only medication related to varicella, herpes zoster or VZV-related complications were included.

Medications were divided in ten groups based on their ATC code: anaesthetics; analgesics; antivirals (local); antivirals (systemic); antibacterials (local); antibacterials (systemic); corticosteroids; antipruritics and emollientia; immunoglobulins; and vaccines (Additional file 3).

### Analysis

Data has been stored and analyzed using Microsoft Access, Microsoft Excel and PASW Statistics 18. Because the age distribution in our database slightly differed from the age distribution of the Dutch population we determined standardized incidence estimates of varicella and herpes zoster when extrapolating our results to the Dutch population. These extrapolated results were established using data from the Central Bureau for Statistics (CBS, www.cbs.nl, 2004–2008). The age groups for varicella incidence and associated complications were defined as 0; 1–4; 5–9 and further 5-year-classes till 20 years and above. For herpes zoster this age group was 0–59; 60–64 and >65-years olds. For calculation of incidence rates 95% upper and lower confidence intervals (CI) were used.

To increase plausibility of causal associations between a complication and a VZV infection the complications rates were corrected for a baseline rate. This baseline rate was determined as the background complication rate for the general population in these age classes. Verruca was selected because verruca is, like herpes zoster and varicella, a viral-induced skin disease generally occurring in the same age classes. Verruca is not associated with (severe) VZV-induced complications and is therefore assumed to be comparable to the general population. The background complication rate was established by completing the same analysis for verruca (ICPC–code S03). The corrected rates were used to differentiate in complication rates between healthy individuals (i.e. verruca patients) and patients (i.e. varicella or HZ patients). Corresponding 95% CI were obtained using the Taylor series linearization method [21]. Statistical significance was defined as p < 0.05.

After this correction for the baseline occurrence of the potential complications certain complications as appendicitis, trombocytopenia, vertigo were excluded as having a causal relationship (p < 0.05) with VZV infection. Complications, known as typical VZV associated complications, like cerebellitis, osteomyelitis, meningitis and sepsis were included. To increase plausibility of causal associations of medication use, also medication use was corrected for a baseline rate of verruca. In general the age specific incidence was calculated per year. The probability of complications was calculated over the years, because of the low sample size of some complications.

## Results

In the total cohort analyzed, including 164,631 (2004) to 166,616 (2008) persons; 5,273 patients had a GP consultation for varicella during the period 2004–2008 (Table 1). During the same period 3,371 patients had a GP consultation for herpes zoster.

**Table 1:** Incidence and number of VZV cases per year

		**Varicella**		**Herpes zoster**	
	**Total patients (n) registred at GP**	**cases (n)**	**incidence per 10,000 population**	**cases (n)**	**Incidence per 10,000 population**
2004	164,631	1,018	49.88	619	45.03
2005	166,576	893	43.77	662	46.99
2006	166,344	1,018	49.20	652	44.67
2007	166,141	1,199	58.26	700	49.67
2008	166,616	1,145	56.60	738	51.10

### Varicella

#### Incidence of varicella GP consultations

**Figure 1:**
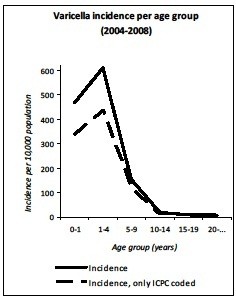
Average age-specific incidence of varicella infection from 2004 to 2008.

**Figure 2:**
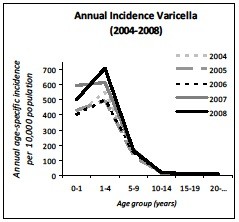
Annual age-specific incidence of varicella infection from 2004 to 2008.

**Figure 3:**
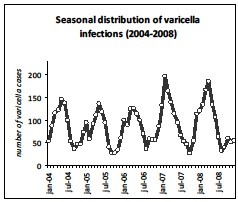
Seasonal distribution of varicella infections by month.

The average of the annual overall incidence of varicella GP consultations in the Netherlands (Figure1 and Table 1), corrected for age distribution of the population, was 51.5 per 10,000 (95% CI 44.4-58.7); 465.5 per 10,000 for 0–1 year-olds; 610.8 per 10,000 for 1–4 year-olds; 153.5 per 10,000 for 5–9 year-olds; 18.0 per 10,000 for 10–14 year-olds; 9.2 per 10,000 for 14–19 year-olds and 7.2 per 10,000 for >20 year-olds (ICPC-coded and free text information). The median age at GP consultations was 3.67 years (IQR: 1.95-5.41). When only ICPC-coded diagnoses were analyzed; the annual overall incidence of GP consultations was 37.7 per 10,000. Of these cases 1.6% was false-positive. The annual age-specific incidence of varicella from 2004 to 2008 shows epidemiological fluctuations (Figure2). The overall standardized annual incidence ranged from 43.8 (2005) to 58.3 per 10,000 (2007) population. As well as the absolute number of varicella cases ranged from 71,375 (2005) to 95,289 (2007). In addition, the incidence of varicella shows a seasonal pattern. In particular, the epidemic season for varicella is late winter/early spring, particularly from February till May (Figure3).

#### Varicella-related complications and symptoms in primary care

During the studied period 5,273 patients with varicella required a GP consultation, of whom 3,901 (74.0%) had any registrated VZV-related complication or symptom. The distinction between the definition of symptoms and complications is addressed in Table 2. Most prevalent symptoms were fever (39.9%), pruritis (27.8%) and coughing (10.5%). (Table 2; note, rates were corrected for the incidence of complications found in verruca patients) Among all varicella patients, 34.9% (n = 1841) consulted a GP for a complication. Upper respiratory tract, ENT or eye complications were found in 28.8% of the cases. Upper respiratory tract infection (13.0%) and otitis media (7.5%) were the most frequent complications. Lower respiratory tract complications occurred in 3.8% of the cases of which 0.6% had pneumonia. Skin infections and cutaneous complications due to varicella occurred in 5.6% of the cases; most frequent complication was impetigo (3.0%). Neurologic complications occurred in 0.9% of the cases, most commonly convulsions (0.7%), but also more serious complications as encephalitis and meningitis were registrated (encephalitis once and meningitis three times during the study period). Serious complications were also seen due to systemic bacterial infections in a small group (n = 8). The occurrence of complications was not age-dependent but varies per complication. Complications as otitis media, bronchitis, convulsions were seen more often in children <4 years of age and pharyngitis, neuralgia and pneumonia more often in >20 year-olds (Table 2; corrected rates). None of the patients died due to varicella infection.

**Table 2:** Varicella-related complication rates in Primary Care

	**Complication rates all ages**			**Complication rates (%) per age group**					**Corrected rates (%)**		
**(n = 5,273)**	**(n)**	**(%)**	**(95% CI)**	**0-4**	**5-9**	**10-19**	**>20**	**All ages**	**(95% CI)**	**<10**	**(95% CI)**
**Upper respiratory tract. ENT complications**											
Conjunctivitis	162	3.07	(2.64-3.57)	3.5	1.9	0.6	2.9	2.08*	(1.58-2.57)	1.98*	(1.38-2.58)
Otitis media	619	11.74	(10.90-12.64)	13.7	8.7	3.6	4.6	7.49*	(6.56-8.42)	7.48*	(6.32-8.64)
Upper respiratory tract infection	942	17.86	(16.85-18.92)	21.0	10.3	3.0	13.5	13.03*	(11.93-14.12)	12.82*	(11.50-14.15)
Lymphadenitis	32	0.61	(0.43-0.86)	0.6	0.9	0.0	0.2	0.51*	(0.29-0.72)	0.57*	(0.32-0.82)
Tonsillitis	219	4.15	(3.65-4.73)	4.4	4.2	3.0	2.2	2.84*	(2.27-3.41)	2.26*	(1.53-2.99)
Pharyngitis	46	0.87	(0.65-1.16)	0.8	0.8	1.2	1.9	0.32*	(0.04-0.60)	0.32	(0.00-0.64)
Sum of complications	2.020	38.31	(37.01-39.63)	44	26.8	11.4	25.3	26.27		25.43	
Total number of patients^a^	1.516	28.75	(27.54-29.99)								
**Lower respiratory tract complications**											
Pneunomia	41	0.78	(0.57-1.05)	0.6	1.1	0.6	1.9	0.63*	(0.39-0.88)	0.49*	(0.23-0.76)
Bronchitis	163	3.09	(2.66-3.59)	3.7	1.8	1.2	1.4	2.26*	(1.77-2.75)	2.41*	(1.83-3.00)
Sum of complications	204	3.87	(3.38-4.42)	4.3	2.9	1.8	3.3	2.89		2.90	
Total number of patients^a^	199	3.77	(3.29-4.32)								
**Skin infectious/cutaneous complications**											
Pyoderma	7	0.13	(0.06-0.27)	0.1	0.0	0.0	0.5	0.05	(−0.06-0.16)	0.11*	(0.01-0.20)
Abscess	19	0.36	(0.23-0.56)	0.3	0.4	0.6	0.7	0.04	(−0.15-0.23)	0.20*	(0.01-0.39)
Skin infection	37	0.70	(0.51-0.97)	0.7	0.8	0.6	0.7	0.52*	(0.28-0.76)	0.63*	(0.38-0.88)
Scar Tissue	53	1.01	(0.77-1.31)	0.8	0.9	2.4	2.6	0.21	(−0.10-0.51)	0.39*	(0.07-0.71)
Impetigo	196	3.72	(3.24-4.26)	4.1	3.4	2.4	1.4	3.04*	(2.51-3.57)	2.49*	(1.82-3.15)
Sum of complications	312	5.92	(5.31-6.59)	6.0	5.5	6.0	5.9	3.86		3.82	
Total number of patients^a^	297	5.63	(5.04-6.29)								
**Neurologic complications**											
Neuralgia	2	0.04	(0.01-0.14)	0.0	0.0	0.0	0.5	0.02	(−0.03-0.08)	0.00	
Meningitis	3	0.06	(0.02-0.17)	0.1	0.0	0.0	0.2	0.05	(−0.02-0.12)	0.04	(−0.02-0.10)
Encephalitis	1	0.02	(0.00-0.11)	0.0	0.1	0.0	0.0	0.02	(−0.02-0.06)	0.02	(−0.02-0.06)
Convulsion	37	0.70	(0.51-0.97)	0.9	0.3	0.0	0.0	0.69*	(0.46-0.91)	0.77*	(0.51-1.02)
Ataxia (Cerebellitis)	1	0.02	(0.00-0.11)	0.0	0.0	0.0	0.0	0.02	(−0.02-0.06)	0.02	(−0.02-0.06)
Movement and stability dysfunction	1	0.02	(0.00-0.11)	0.0	0.0	0.0	0.2	0.02	(−0.02-0.06)	0.00	(0.00-0.00)
Facial Palsy	2	0.04	(0.01-0.14)	0.03	0.0	0.0	0.2	0.04	(−0.01-0.09)	0.02	(−0.02-0.06)
Coma	1	0.02	(0.00-0.11)	0.0	0.0	0.6	0.0	0.02	(−0.02-0.06)	0.00	(0.00-0.00)
Sum of complications	48	0.91	(0.69-1.20)	1.0	0.4	0.6	1.1	0.88		0.87	
Total number of patients^a^	48	0.91	(0.69-1.20)								
**Gastrointestinal tract complications**											
Stomatitis	16	0.30	(0.19-0.49)	0.4	0.2	0.0	0.2	0.27*	(0.12-0.43)	0.27*	(0.10-0.45)
Gastroenteritis	125	2.37	(1.99-2.82)	2.8	1.5	0.0	1.7	1.46*	(1.84-2.67)	1.60*	(1.07-2.13)
Sum of complications	141	2.67	(2.27-3.15)	3.2	1.7	0	1.9	1.73		1.87	
Total number of patients^a^	139	2.64	(2.24-3.10)								
**Complications due to (systemic) bacterial infections**											
Sepsis	4	0.08	(0.03-0.19)	0.1	0.1	0.0	0.0	0.07	(−0.01-0.14)	0.09	(0.00-0.17)
Osteomyelitis	1	0.02	(0.00-0.11)	0.03	0.0	0.0	0.0	0.02	(−0.02-0.06)	0.02	(−0.02-0.06)
Pyogen arthritis	3	0.06	(0.02-0.17)	0.03	0.0	0.0	0.5	0.06	(−0.01-0.12)	0.02	(−0.02-0.06)
Sum of complications	8	0.15	(0.08-0.30)	0.2	0.1	0.0	0.5	0.15		0.13	
Total number of patients^a^	8	0.15	(0.08-0.30)								
**Symptoms**											
Mouth blisters	55	1.04	(0.80-1.36)	0.9	1.1	1.8	1.4	0.98*	(0.70-1.26)	0.86*	(0.56-1.16)
Coughing	757	14.36	(13.44-15.33)	17.0	8.7	1.8	9.4	10.50*	(9.50-11.50)	10.83*	(9.63-12.03)
Snivelling	237	4.49	(3.97-5.09)	6.1	0.7	0.6	0.5	4.18*	(3.62-4.75)	4.45*	(3.79-5.11)
Fever	2.550	48.36	(47.01-49.71)	51.5	40.2	37.0	44.0	39.91*	(38.48-41.34)	37.96*	(36.25-39.68)
Fatigue	401	7.60	(6.92-8.35)	9.3	3.9	3.0	3.4	6.02*	(5.27-6.76)	7.02*	(6.17-7.86)
Emesis/Vomiting	417	7.91	(7.21-8.67)	9.1	5.1	6.1	4.3	6.47*	(5.72-7.23)	6.34*	(5.45-7.24)
Problems Eating/drinking	664	12.59	(11.72-13.52)	15.2	7.1	6.1	4.8	10.73*	(9.81-11.65)	11.75*	(10.70-12.81)
Pruritis/Itching	1.845	34.99	(33.71-36.29)	32.1	40.0	44.2	45.2	27.82*	(26.46-29.17)	28.11*	(26.59-29.63)
Skin rash/Exanthema	439	8.33	(7.61-9.10)	8.2	6.8	12.7	11.3	7.46*	(6.69-8.22)	7.02*	(6.19-7.84)
Dehydration/Diarreah	433	8.21	(7.50-8.98)	10.0	4.5	3.0	2.9	6.72*	(5.95-7.49)	7.04*	(6.13-7.94)

^a^The total number of patients may be less than the total sum of complications because some patients had more than one complication in this group.

* = p < 0.05.

#### Varicella incidence and complications requiring hospital care

The incidence of consultation for varicella in hospital care, corrected for age distribution of the population, was 0.86 per 10,000. The overall referral rate based on GP consultations among all varicella cases was 1.7%. For varicella 89 cases were referred to the hospital for complications associated with VZV infection. The median age of the cases was 2.28 years (IQR: 0.96-4.01). Most cases (69%) visited a pediatrician and 19% of the cases went to the emergency care. Most common complications in hospital care were dehydration (14.6%); skin super infections (18%) and pneumonia (12.4%) (Table 3).

**Table 3:** VZV complications in Hospital Care

**Varicella (n = 89)**	**n**	**(%)**	**Herpes zoster (n = 119)**	**n**	**(%)**
**Neurologic complications**			**Neurologic complications**		
Meningitis	1	(1.1%)	Meningitis	1	(0.8%)
Cerebrellitis	1	(1.1%)	Cerebellitus	1	(0.8%)
Bells palsy	1	(1.1%)	Postherpetic neuralgia	4	(3.4%)
Convulsions	7	(7.9%)	Trigeminal neuralgia	3	(2.5%)
Headache	1	(1.1%)	Bells palsy	1	(0.8%)
Ataxia	1	(1.1%)	Facial palsy	7	(5.9%)
**Cutaneous complications**			Neurological pain	10	(8.4%)
Skin superinfections	16	(18%)	Herpes zoster oticus	1	(0.8%)
Cellulitis	2	(2.2%)	**Ocular complications**		
Phlegmon	1	(1.1%)	Herpes zoster ophthalmicus (not specified)	20	(16.8%)
Abscess	1	(1.1%)	Conjunctivitis	3	(2.5%)
Impetigo (secondary)	2	(2.2%)	Keratitis	4	(3.4%)
Scar tissue	3	(3.4%)	Visus	5	(4.2%)
**Complications due to systemic bacterial infections**			**Cutaneous complications**		
Osteomyelitis	1	(1.1%)	Herpes zoster dermatome	45	(37.8%)
Pyogen artritis	1	(1.1%)	Cellulitis	1	(0.8%)
Sepsis	1	(1.1%)	Pyoderm	1	(0.8%)
**Respiratory tract complications**			Cutaneous pain	1	(0.8%)
Pneumonia	11	(12.4%)	Skin superinfections	2	(1.7%)
Bronchitis (acute)	1	(1.1%)	**Other complications**		
Upper resperatory tract infection (not specified)	2	(2.2%)	Pneumonia	5	(4.2%)
Tonsillitis	2	(2.2%)	Thrombocytopenia	1	(0.8%)
Lymphadenitis	3	(3.4%)	Sepsis	1	(0.8%)
Pharyngitis	1	(1.1%)			
**Other complications**					
Stomatitis	3	(3.4%)			
Hemorrhagic varicella	1	(1.1%)			
Fever (extremely high temperature)	3	(3.4%)			
Dehydration	13	(14.6%)			
Thrombocytopenia	1	(1.1%)			
Anemia	1	(1.1%)			
Extreme varicella (not specified/unknown complications)	5	(5.6%)			

* a case can have more than one complication.

#### Treatment of varicella infections

Approximately 47.7% of the patients received medication for varicella or associated complication (Table 4). For varicella less than 1% of the cases received antiviral therapy while 11.2% of the cases used antibacterials. Most common therapy for varicella was based avoiding itching. Hence, 13.5% of the cases received aneasthetics and 11.3% antipruritics and emollientia. VZV immunoglobulins were prescribed in three cases. Some medication gave a negative corrected rate, analgesics and local antivirals, as the baseline rate was higher then the percentage of use among varicella patients, suggesting no association with varicella.

**Table 4:** VZV-related treatment

	**Varicella**				**Herpes zoster**			
**Medication**	**Prescriptions**	**Varicella cases**		**Corrected**	**Prescriptions**	**HZ cases**		**Corrected**
	**n**	**n**	**(%)**	**(%)**	**n**	**n**	**(%)**	**(%)**
Anaesthetics	925	831	(15.8%)	13.5% *	518	364	(10.8%)	6.4%*
Analgesics	124	110	(2.1%)	−1.5%	1.097	577	(17.1%)	13.5%*
Antivirals (local)	17	17	(0.3%)	−1.2%	67	58	(1.7%)	0.1%
Antivirals (systemic)	24	21	(0.4%)	0.3%*	864	748	(22.2%)	22.0%*
Antibacterials (local)	588	487	(9.2%)	6.7%*	594	453	(13.4%)	10.9%*
Antibacterials (systemic)	678	568	(10.8%)	4.4%*	449	354	(10.5%)	4.5%*
Corticosteroids	394	316	(6.0%)	0.3%	372	316	(9.3%)	0.9%
Antipruritics and emollientia	1.136	1.004	(19.0%)	11.3%*	761	646	(19.2%)	11.5%*
Immunoglobulins	3	3	(0.1%)	0.1%	1	1	(0.03%)	0.03%
Vaccines	0	0	(0.0%)	0.0%	0	0	(0.00%)	0.0%
**Total (any medication)**	**3.889**	**2.516**	**(47.7%)**		**4.723**	**2.286**	**(67.8%)**	

* = p < 0.05.

### Herpes zoster

#### Incidence of herpes zoster GP consultations

**Figure 4:**
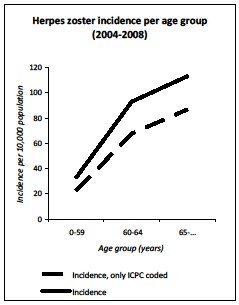
Average age-specific incidence of herpes zoster infection from 2004 to 2008.

**Figure 5:**
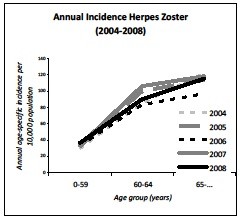
Annual age-specific incidence of herpes zoster infection from 2004 to 2008.

The average annual number of herpes zoster GP consultations in the Netherlands, corrected for age distribution of the population, was 47.5 per 10,000 (95% CI 40.6-54.4). The incidence increased with age; 32.8 per 10,000 for <60 year-olds; 93.1 per 10,000 for 60–64 year-olds and 113.2 per 10,000 for >65 year olds (ICPC-coded and free text information) (Figure4). The median age was 48.51 years (IQR: 29.37-62.41). The incidence ranged from 44.7 (2006) to 51.1 (2008) per 10,000 population, annually (Figure5). Furthermore, the absolute number of herpes zoster cases raged from 72,969 (2006) to 83,828 (2008). If the herpes zoster incidence was estimated on ICPC-code only, the incidence was 34.0 per 10,000.

#### Herpes zoster-related complications and symptoms

Of the 3,371 herpes zoster cases identified, 2,155 (63.9%) had any complication or symptom. Among the zoster cases, we found a complication rate of 32.9% (n = 1,108). The distinction between the definition of symptoms and complications is addressed in Table 5. Common symptoms were pruritis (22.6%), fever (12.4%) and skin rash (11.0%).

**Table 5:** Herpes zoster- related complication rates in Primary Care

**(n = 3.371)**	**Complication rates all ages**		**Complication rates (%) per age group**					**Corrected rate (%)**		
	**(n)**	**(%)**	**(95% CI)**	**<60**	**60-64**	**>65**	**All**	**(95% CI)**	**>60**	**(95% CI)**
**Upper respiratory tract. ENT complications**										
Otitis media	203	6.02	(5.27-6.88)	6.0	5.2	6.3	1.77*	(0.90-2.64)	3.55*	(1.73-5.37)
Upper respiratory tract infection	350	10.38	(9.40-11.46)	8.9	12.3	14.7	5.55*	(4.45-6.63)	7.96*	(5.24-10.69)
Sum of complications	553	16.40	(15.19-17.69)	14.9	17.5	21.0	7.32		11.51	
Total number of patients^a^	501	14.86	(13.70-16.10)							
**Eye complications**										
Keratitis	13	0.39	(0.23-0.66)	0.3	0.0	0.8	0.39*	(0.18-0.59)	0.64*	(0.13-1.16)
Conjunctivitis	48	1.42	(1.08-1.88)	1.4	2.4	1.4	1.42*	(1.02-1.82)	1.08*	(0.14-2.01)
Problems Eyelids	96	2.85	(2.34-3.47)	2.5	4.7	3.6	2.83*	(2.26-3.39)	3.76*	(2.50-5.02)
Visus	18	0.53	(0.34-0.84)	0.4	0.9	0.8	0.53*	(0.29-0.78)	0.86*	(0.27-1.45)
Sum of complications	175	5.19	(4.49-5.99)	4.6	8.0	6.6	5.17		6.34	
Total number of patients^a^	147	4.36	(3.72-5.10)							
**Lower respiratory tract complications**										
Pneunomia	15	0.44	(0.27-0.73)	0.2	0.9	1.0	0.30*	(0.07-0.53)	0.65	(−0.08-1.37)
Bronchitis	50	1.48	(1.13-1.95)	1.3	2.8	1.8	0.65*	(0.21-1.08)	0.21	(−1.53-1.11)
Sum of complications	65	1.93	(1.52-2.45)	1.5	3.7	2.8	0.95		0.86	
Total number of patients^a^	64	1.90	(1.49 2.42)							
**Skin infectious/cutaneous complications**										
Phlegmon	13	0.39	(0.23-0.66)	0.4	0.0	0.6	0.29*	(0.08-0.51)	0.22	(−0.30-0.73)
Pyoderma	13	0.39	(0.23-0.66)	0.4	0.0	0.4	0.30*	(0.08-0.51)	0.11	(−0.36-0.58)
Abscess	30	0.89	(0.62-1.27)	0.8	1.9	0.8	0.57*	(0.24-0.90)	0.86*	(0.13-1.59)
Skin infection	32	0.95	(0.67-1.34)	0.9	0.0	1.4	0.77*	(0.43-1.10)	0.86*	(0.13-1.59)
Cellulitis	21	0.62	(0.41-0.95)	0.6	1.4	0.4	0.55*	(0.28-0.82)	0.43	(−0.16-1.02)
Scar Tissue	46	1.36	(1.02-1.82)	1.3	0.5	1.9	0.57*	(0.15-0.99)	0.97*	(0.01-1.93)
Impetigo	112	3.32	(2.77-3.98)	3.9	1.4	1.8	2.65*	(2.03-3.27)	1.61*	(0.75-2.47)
Sum of complications	267	7.92	(7.06-8.88)	8.3	5.2	7.3	5.7		5.06	
Total number of patients^a^	234	6.94	(6.13 7.85)							
**PHN**										
PHN	195	5.78	(5.05 6.62)	3.4	8.0	13.1	5.78*	(5.00-6.57)	11.92*	(9.84-14.00)
**Neurologic complications other than PHN**										
Syncope	27	0.80	(0.55-1.16)	0.5	0.5	1.8	0.38*	(0.06-0.70)	1.50*	(0.72-2.29)
Neuralgia (Trigeminal)	75	2.22	(1.78-2.78)	1.8	2.8	3.5	2.21*	(1.71-2.71)	3.33*	(2.18-4.48)
Meningitis	2	0.15	(0.02-0.22)	0.2	0.0	0.1	0.14	(−0.03-0.14)	0.11	(−0.10-0.32)
Ataxia (Cerebellitis)	1	0.03	(0.01-0.17)	0.04	0.0	0.0	0.03	(−0.03-0.09)	0.00	
Movement and stability dysfunct.	6	0.18	(0.08-0.39)	0.1	0.0	0.6	0.18*	(0.04-0.32)	0.43*	(0.01-0.85)
Vertigo	97	2.88	(2.36-3.50)	2.3	1.9	5.0	2.36*	(1.79-2.94)	3.12*	(1.64-4.59)
Facial palsy	33	0.98	(0.70-1.37)	0.8	0.0	1.8	0.98*	(0.65-1.31)	1.40*	(0.64-2.15)
Coma	1	0.03	(0.01-0.17)	0.0	0.0	0.0	0.03	(−0.03-0.09)	0.00	
Pain face	24	0.71	(0.48-1.06)	0.5	0.9	1.3	0.69*	(0.41-0.97)	1.18*	(0.49-1.88)
Sum of complications	266	7.89	(7.03-8.85)	6.24	6.1	14.1	7.00		11.07	
Total number of patients^a^	258	7.65	(6.80-8.60)							
**Gastrointestinal tract complications**										
Stomatitis	9	0.27	(0.14-0.51)	0.2	0.5	0.3	0.24*	(0.06-0.41)	0.43*	(0.01-0.85)
Gastroenteritis	52	1.54	(1.18-2.02)	1.2	3.3	2.2	0.63*	(0.18-1.07)	1.18	(−0.05-2.42)
Pancreatitis	10	0.30	(0.16-0.55)	0.1	1.9	0.6	0.25*	(0.07-0.44)	0.86*	(0.27-1.45)
Sum of complications	71	2.11	(1.67-2.65)	1.5	5.7	3.1	1.12		2.47	
Total number of patients^a^	66	1.96	(1.54-2.48)							
**Complications due to (systemic) bacterial infections**										
Sepsis	1	0.03	(0.01-0.17)	0.04	0.0	0.0	0.02	(−0.04-0.08)	0.00	
Pyogen arthritis	3	0.09	(0.03-0.26)	0.08	0.5	0.0	0.09	(−0.01-0.19)	0.11	(−0.10-0.32)
Sum of complications	4	0.12	(0.05-0.30)	0.1	0.5	0.0	0.11		0.11	
Total number of patients^a^	4	0.12	(0.05-0.30)							
**Symptoms**										
Coughing	241	7.15	(6.33-8.07)	6.1	7.5	10.7	3.29*	(2.36-4.22)	6.88*	(4.66-9.11)
Snivelling	26	0.77	(0.53-1.13)	0.7	0.5	1.0	0.46*	(0.15-0.77)	0.54	(−0.16-1.23)
Fever	703	20.85	(19.52-22.26)	20.5	20.8	22.0	12.41*	(10.95-13.85)	16.23*	(13.21-19.25)
Fatigue	148	4.39	(3.75-5.14)	4.2	1.9	5.8	2.80*	(2.08-3.52)	3.23*	(1.60-4.85)
Emesis/Vomiting	121	3.59	(3.01-4.27)	3.0	3.3	5.6	2.15*	(1.49-2.81)	4.30*	(2.79-5.81)
Pruritis/Itching	1.005	29.81	(28.29-31.38)	31.7	17.9	26.8	22.64*	(21.03-24.23)	17.20*	(13.95-20.46)
Skin rash/Exanthema	400	11.87	(10.82-13.00)	11.2	13.2	13.6	11.00*	(9.89-12.09)	12.78*	(10.52-15.05)
Dehydration/Diarreah	106	3.14	(2.61-3.79)	2.6	2.4	5.3	1.58*	(1.03-2.27)	3.76*	(2.29-5.23)

^a^The total number of patients may be less than the total sum of complications because some patients had more than one complication in this group.

* = p < 0.05.

Neurological complications were found in 13.5% of the cases. Post-herpetic neuralgia (5.8%) and trigeminal neuralgia (2.2%) were the most frequent neurological complications. Fifteen percent of the cases had an upper respiratory tract or ENT complication. Complications associated with herpes zoster were ocular complications (4.4%) and lower respiratory tract infections (1.9%). Approximately 6.9% of the cases develop cutaneous complications, most common impetigo (2.7%). Serious systemic bacterial complications as sepsis and pyogen arthritis were found in 0.1% of the cases. Three cases died within one month after herpes zoster consultation (Table 5).

#### Herpes zoster incidence and complications in hospital care

There were 119 herpes zoster related hospital referrals, i.e. 1.55 per 10,000. The overall referral rate based on GP consultations among all cases was 3.5%. The median age of the cases was 52.8 years (IQR: 40.72-66.91). More than 35% of the cases were seen by a dermatologist; 23% by an ophthalmologist and 16% by a neurologist. Most common complications were herpes zoster dermatomal rash (37.8%); herpes zoster ophthalmicus (27.7%) and neurological pain (8.4%) (Table 3).

#### Treatment of herpes zoster infections

Approximately 67.8% of the herpes zoster cases received medication (Table 4). Almost one-quarter of the patients received antiviral therapy, most systemic antivirals (22.0%). More than 15% of the cases received antibacterials, most often local antibacterials (10.3%). Of the patients, 11.5% received antipruritics and emollientia and 6.4% used aneasthetics. Analgesics were used in 13.5% of the cases.

## Discussion

We used data from a research database from the municipality of Almere, combining GP, hospital data and pharmacy data to analyze the epidemiologic characteristics and disease burden of varicella and herpes zoster in the Netherlands. This study shows that the incidence of GP-diagnosed VZV infections is much higher than expected based on ICPC-codes solely. Because the use of both, coded and non-coded information, the incidences found in present study provide updated data on the number of people with VZV complaints that prompt patients to seek medical care.

### Varicella

To our knowledge, the current study is the first varicella study based on ICPC-coded and non-coded information. Our data confirms the burden of varicella as a common disease which affects a large proportion of the population each year, as demonstrated by the high overall standardized annual GP-incidence rate of 51.5 per 10,000. When including free text in this analysis the incidence of varicella is over twice as high when compared to the national registration system. When the analyses were restricted to coded information the national registration system registrated an incidence of 22.2 per 10,000 based on coded information for varicella (2004–2008) [22] whereas, the incidence determined in ZGA was higher, namely 37.7 per 10,000 over the same period. Currently Dutch epidemiological data may not accurately reflect the true burden of varicella due to under-reporting to statutory notification systems. Underestimation of national registration is not unusual, it is shown before in the Netherlands and abroad [6,23,24] for VZV infections and other diseases [25]. Our overall incidence of VZV-related GP consultations is comparable to England and Wales, where the incidence is approximately 50.7 per 10,000 [26].

Most people in the Netherlands will have contracted varicella, before the age of five [1]. Our data show a comparable age-specific disease distribution to a study about Dutch VZV-seroprevalence, where 93% of the people already contract varicella before the age of five [1]. Compared to other countries the mean age of infection in the Netherlands is rather low [27]. Yet, there are studies that show a decrease in the peak age of varicella in England. Between 1983 and 1998 the incidence rates for varicella fell in children aged 5–14 years old (by about 50%), but doubled in children aged 0–4 years [28].

Despite public perception of varicella infection being a harmless childhood disease, different complications can occur [29]. The most common complications associated with varicella in primary care are upper respiratory tract infections, otitis media and impetigo. Complications were not limited to infections beyond adolescence, as forthcoming research has showed [30,31]. Contrary to common belief some complications have the tendency to decrease with age while other complications increase, i.e. age seems not the only factor that drives complications associated with VZV. In the majority of cases varicella is a self-limiting disease and usually no specific treatment is required. Nevertheless, 34% of the varicella patients seeking GP consultation develop an associated complication and nearly half of the patients who consulted the GP received medication, mostly antibacterials. Based on the results presented in this study we estimate that between 3 to 8% of all Dutch patients with varicella, depending on age, consult a GP due to a complication. Our findings are similar to data from Germany [32], France [33] and the United States of America [34], were it is estimated that in approximately 2 to 6% of cases attending a general practice. Furthermore of these varicella patients 1.7% experiences complications severe enough to seek hospital care. The incidence of hospital admission was 0.86 per 10,000, which is comparable with recently published Dutch hospitalization data [23]. Though these percentages are low they portray proportions of approximately the entire population, e.g. fractions of all patients with a varicella infection.

### Herpes zoster

While varicella is a common childhood disease, herpes zoster occurs with increasing frequency with increasing age [5]. The incidences found in the present study increased with age: 32.8 per 10,000 for <60 year-olds, 93.1 per 10,000 for 60-64-year-olds and 113.2 per 10,000 for people above 65-years; with an overall standardized annual incidence of 47.5 per 10,000 (95% CI 40.6-54.4). This is comparable with incidences found in United Kingdom [35] and United States [36] in this period. Our overall incidence is higher than the Dutch registration of 32.5 per 10,000 (1998–2001) [1] but our incidence based on coded information only is nearly compatible with Dutch registry, namely 34.0 per 10,000. This incidence is similar to a previous Dutch study [6] and data from the Belgium sentinel system [37]. The incidence of consultation in hospital care is 1.55 per 10,000 for herpes zoster, which is comparable with incidences in some other European countries [37,38]. However the incidence is much higher then previously described in the Netherlands [1] and in England and Wales [39]. Associated morbidity is mostly age dependent; it increased during life-time. For example the incidence rate of PHN, VZV most registrated complication, is low (3.4%) in patient younger than 60 year and 13.9% in patient older than 65 years of age. It is believed that herpes zoster is generally associated with normal aging and with anything that causes reduced immunocompetence [40].

The estimated complication rate was 32.9%, most complications corresponded to the affected dermatome, for example trigeminal neuralgia. More than two-third of the cases who consulted a GP for herpes zoster received medication. Almost one-quarter of all herpes zoster patients were treated with antiviral therapy, which is comparable to the 22.5% found in a Dutch GP-survey [41].

### Strength and limitations

The scope of this study was limited to patients with varicella and herpes zoster seeking help from a general practitioner. In particular, the study relies on what GPs document with ICPC-coding and in free text which may not be totally complete. Even though the database was very elaborate, the data included for hospital care (referrals/discharge) was limited to the referrals recorded by the attending GP and might underestimate the occurrence of more serious complications.

Nevertheless, false negative and positive misclassification may have occurred. We relied on GP diagnoses which are usually based on the recording of symptoms without objective diagnostic evidence. Moreover, we may have missed VZV cases that did not fall within the range of search terms included in our study although all synonyms and lay names were generated by Dutch experts. However, due to our elaborate search the underestimation following this possible bias is expected to be very small. Misclassification can also appear by the occurrence of VZV associated complications. Because most potential VZV associated complications are not complications caused only specific by VZV, we can not consider all relationships within our chosen time range equally plausible in a causal sense. For instance, it is not clear if the herpes zoster related deaths were caused by herpes zoster or if herpes zoster was a reactivated because of reduced immunocompetence of the patients. To increase plausibility of causal associations the complication rates were corrected for a baseline rate by using a reference disease verruca (warts). Verruca is, like herpes zoster and varicella, caused by a virus and affects the skin. A possible limitation of our study is that some of the patients might already have been treated before 2004 or continued treatment in 2009. Therefore we might have an underestimation of cases and health care resource use. The projections of incidences on the general Dutch population are based on an assumption of demographic generalisability of the study population. Possibly, differences in population characteristics such as ethnicity and socio-economic factors may explain the observed difference in incidence rates. More likely, however, would be that the differences with the national registration (NIVEL) are related to differences in exact case definitions and our extensive validation process. Because the age distribution in our database slightly differed from the age distribution of the Dutch population we determined standardized incidence estimates of varicella and herpes zoster when extrapolating our results to the Dutch population.

The strength of this study lies in the elaborate information available in the research database from the municipality of Almere which combines GP data (incl. free text in the medical record notes), hospital data and pharmacy data to analyze epidemiologic characteristics and disease burden of varicella and herpes zoster in the Netherlands. Another strength of this study lies in the use of both coded and non-coded information; this way our study is based on a broader case definition enabling us to avoid possible underestimation by missing cases only reported in free text field. The incidence estimations were based on a sensitive and detailed search algorithm (including typing errors and misspellings) followed by a review of the full-text medical records of the selected patients. In combination, this ZGA data enables us to have an extensive view of the occurrence of VZV and it’s complications in the GP practices.

## Conclusion

This study has provided important epidemiological background data on VZV-infections in the Netherlands, which is useful for clinical practice and decisions makers.

Present study shows the true burden of disease due to a primary or recurrent VZV infection based on coded and non coded information. Though the proportion of people that undergo complications following VZV infections is small, almost the entire population gets primary VZV-infection. Therefore, the absolute number of people with a complication for varicella is high. Our study shows that the occurrence of complications is not age-dependant but varies per complication. This challenges the common belief that (severe) complications only occur when getting varicella at an older age. Frequency of reactivation of the virus occurs with advancing age. Associated morbidity increases also during life-time. Although the risk of suffering herpes zoster infection over the course of life-time has previously been estimated at 20 at 30% [5], the health care resource uses are as high as for varicella.

It is important to know that when incidences of VZV-infections and their associated complications are estimated based on coded information only, one should be aware of a gross underestimation of the incidences. Our analysis may have important implications for common practice concerning VZV patients. Additionally policymakers may take our results into consideration when discussing the prevention of VZV-associated complications. More insights regarding medical complications resulting into hospitalization and intramural treatment are necessary to get a better understanding of disease burden.

### Ethical approval

The study was approved by the scientific council of the Primary Health Care Organisation Almere. Anonymous patient data were used; therefore, no ethical approval was necessary for this project.

## Competing interest

PDG and SACF are employees of the scientific department of GlaxoSmithKline B.V. in the Netherlands. GlaxoSmithKline is a manufacturer of a varicella vaccine. MJP has received research grants from various pharmaceutical industries to study cost-effectiveness of vaccines. The other authors declare that they have no competing interests.

## Authors’ contributions

PDG, SACF and JGJP designed the study. Data collection was performed by JGJP under supervision of PCES. Data analyses were performed by JGJP under supervision of MJP. JGJP, PDG and SACF drafted the manuscript. PCES and MJP commented on the drafts and advised in various stages of the research. All authors have been involved in drafting the manuscript or revising it critically for important intellectual content, and have given final approval of the version to be published.

## Supplementary Material

Additional file 1 **APPENDIX A.** Search terms for varicella-related complications and symptoms. Click here for file

Additional file 2 **APPENDIX B.** Search terms for herpes zoster-related complications and symptoms. Click here for file

Additional file 3 **APPENDIX C.** Search Terms for VZV-related medication. Click here for file
