# Supplementary material for: Epidemiological characteristics and societal burden of varicella zoster virus in the Netherlands
Source: BMC Infect Dis. 2012 May 10;12:110. doi: 10.1186/1471-2334-12-110 (PMC3464966; doi:10.1186/1471-2334-12-110)
Supplement: Additional file 2 — APPENDIX B. Search terms for herpes zoster-related complications and symptoms. [file 1471-2334-12-110-S2.pdf]

APPENDIX: Search terms for herpes zoster complications

APPENDIX B: Search terms for herpes zoster-related complications and symptoms		
	ICPC code	Free text (synonyms. medical or lay names with or without grammatical mistakes of the following wordings)
**Upper respiratory tract, ENT complications**		
Otitis media	H71/H72	Otitis media (OME or OMA); middle ear infection/inflammation
Upper respiratory tract infection	R74	Upper respiratory tract infections *(*URTI or URI)
Lymphadenitis	B70/B71	Lymphadenitis; inflammation/infection of the lymph node
Tonsillitis	R76	Tonsillitis; inflammation/infection of the tonsils; angina
Pharyngitis	R74.02	Pharyngitis; inflammation/infection of the throat or pharynx
**Eye complications**		
Conjunctivitis	F70	Conjunctivitis; inflammation of the eye
Keratitis	F73.2	Keratitis; inflammation of the cornea
Visus	F05	Visus. visual perception problems; Visual disturbance
Eyelid problems	F16	Problems of the eyelid; Eyelid symptom/complaint
**Lower respiratory tract complications**		
Pneumonia	R81	Pneumonia; infection/inflammation of the lung
Bronchitis	R78	Bronchitis
**Skin infectious/cutaneous complications**		
Phlegmon		Phlegmon; flegmon; infection with streptococci of soft tissue
Pyoderma	S76	Pyoderma (gangrenosum) (PG); pus in the skin
Abscess	S10 / D95.2	Abscess; Furuncle
Skin infection	S11	Skin infection; (secondary) inflammation/infection of the skin
Cellulitis	S10.3	Cellulitis; bacterial infection of the deeper layers of the skin
Scar Tissue		Scar Tissue
Impetigo	S84	Impetigo (*in Dutch krentenbaard*)
**PHN**		
PHN	S70.2	Post herpetic neuralgia (PHN)
**Neurologic complications. other than PHN**		
Syncope	A06	Syncope; collapse; loss of consciousness
Neuralgia (Trigiminus)	N92	Neuralgia; pain in a nerve pathway
Meningitis	N71.01/N71.02	Meningitis; inflammation of the meninges *(in Dutch nekkramp)*
Encephalitis	N71.03	Encephalitis; acute inflammation of the brain
Convulsion	N07	Convulsion; seizure *(in Dutch (koorts)stuipen*)
Ataxia (Cerebellitis)	N29	Ataxia: dysfunction of the cerebellum
Movement and stability dysfunction (others than ataxia)	N06	Movement and stability dysfunction
Vertigo	N17	Vertigo; dizziness
Facial palsy	N91	Facial(is) paresis; Facial paralysis/palsy
Pain face	N03	Pain face
Coma	A07	Coma; unconsciousness
Reye's syndrome		Reye's syndrome
**Gastrointestinal tract complications**		
Stomatitis	D83	Stomatitis; viral infection of the mouth
Gastroenteritis	D73	Gastroenteritis (GE); gastric flu; inflammation of the gastrointestinal tract
Pancreatitis	D99.04	Pancreatitis; inflammation of the pancreas
Appendicitis	D88	Appendicitis; inflammation of the appendix
**Haematological complications and coagulation disorders**		
Thrombocytopenia	B83.02	Thrombocytopenia; reduced platelet (thrombocyte)
Haemorrhage	A10	Haemorrhage; bleeding; loss of blood
Coagulation	B83	Coagulation: formation of a blood clot (problems)
**Complications due to (systemic) bacterial infections**		
Sepsis	W70	Sepsis*;* Blood Infection
Osteomyelitis	L70.01	Osteomyelitis; inflammation of the bone
Pyogen arthritis	L70.02	(pyogen) arthritis; arthri*t*is caused by Streptococcus pyogenes
Necrotizing fasciitis		Necrotizing fasciitis; infection of soft tissue
**Death**		
Death	A96	Death; deceased
**Symptoms**		
Mouth blisters	D83.02	Mouth blister *(in Dutch aften)*
Coughing	R05	Cough; sputum
Snivelling	A15	Snivelling; whining
Fever	A03	Fever; pyrexia; temperature rises
Fatigue	A04	Fatigue; exhaustion; listlessness
Problems Sleeping		Problems with sleeping
Emesis/Vomiting	D10	Emisis; Vomiting
Problems Eating/drinking	T03/T04	Problems Eating; Problems drinking; feeding problems
Pruritis	S02	Pruritis; Itching

Exanthema	S06/S07	Skin rash; exanthema; exanthem
Headache	N01	Headache; pain or discomfort in the head. scalp. or neck
Dehydration/Diarrhea	D11/ D70/ T11	Dehydration; Diarrhea; loss of body fluids
