# Supplementary material for: Epidemiological characteristics and societal burden of varicella zoster virus in the Netherlands
Source: BMC Infect Dis. 2012 May 10;12:110. doi: 10.1186/1471-2334-12-110 (PMC3464966; doi:10.1186/1471-2334-12-110)
Supplement: Additional file 3 — APPENDIX C. Search Terms for VZV-related medication. [file 1471-2334-12-110-S3.pdf]

**Appendix: Search terms for VZV-related medication**

| APPENDIX C: Search Terms for VZV-related medication | | |
|---|---|---|
| **Medication group** | **ATC code** | **Definition** |
| Anaesthetics | N01 | Anaesthetics |
| Analgetics | N02 | Analgetics |
| Antivirals (local) | D06BB<br>S01A<br>S02A | Antivirals (dermatological use)<br>Ophthalmologicals (antiviral)<br>Otologicals (antiviral) |
| Antivirals (systemic) | J05 | Antivirals for systemic use |
| Antibacterials (local) | D06A<br><br>D06C<br><br><br>S01B<br>S02A | Antibiotics for topical use (dermatological use)<br>Antibiotics and chemotherapeutics. combinations(dermatological use)<br>Ophthalmologicals (antibacterial)<br>Otologicals( antibacterial) |
| Antibacterials (systemic) | J01 | Antibacterials for systemic use |
| | J04 | Antimycobacterial |
| Corticosteroids | D07 | Corticosteroids. dermatological preparations |
| Antipruritics and emollientia | D04<br><br>D02A | Antipruritics. including antihistamines., anesthetics. etc<br>Emollientia en protectiva |
| Immunoglobulins | J06 | Immune sera and immunoglobulins ( VZV-related) |
| Vaccines | J07B | Vaccines (VZV-related) |
